# Asthma diagnosis and treatment – 1015. Improvements in lung function in an indian population with IgE mediated asthma receiving omalizumab in a real-world setting

**DOI:** 10.1186/1939-4551-6-S1-P15

**Published:** 2013-04-23

**Authors:** Randeep Guleria, Deepak Talwar, Ashok Mahashur, Mangala Kotnis

**Affiliations:** 1 Department of Pulmonary Medicine and Sleep Disorders, All India Institute of Medical Sciences, India; 2 Chest Medicine, Metro Hospital, Noida, India; 3 Chest Medicine, P.D.Hinduja National Hospital, India; 4 Medical Advisor, Novartis India Limited, India

## Background

Omalizumab is a recombinant humanized anti-IgE monoclonal antibody, indicated for add-on therapy for moderate- to severe-persistent allergic (IgE-mediated) asthma. Interim results at the 16 weeks time point of this 52 week study have previously been presented. We present a subgroup analysis looking at the efficacy of omalizumab at 16 weeks in relation to the serum IgE levels at baseline.

## Methods

This open-label, multi-center, observational, prospective study, recruited 72 patients with moderate-to-severe persistent allergic asthma. Clinical efficacy was evaluated on the basis of improvement in asthma exacerbations, days missed at work/college, hospitalizations, ACQ 5, ACT scores, and FEV1. Safety and tolerability were also assessed. Qualitative and quantitative variables are analyzed using Chi-Square tests and paired t-tests, respectively. All parameters are comparing results at week 16 of omalizumab treatment with those at baseline.

## Results

Mean serum-IgE levels at baseline were 437.45 kIU L–1 (range 32-1599 kIU L–1). For analysis purposes patients were categorized into three groups, according to their baseline serum-IgE levels. Group 1 (n=31), comprised patients with baseline serum-IgE levels of 32-250 kIU L–1), and in group 2 (n=24) and 3 (n=17), patients with baseline serum-IgE levels of 251-600 and >600 kIU L–1; respectively. Mean (SD) age in three groups was 53.5 (9.6), 43.8 (15.3) and 60.5 (13.5) years; correspondingly. In group 1, FEV1 levels improved by 0.27 liters (0.88 vs. 1.15, 95%CI ; p=0.000) and in group 2 and 3 by 0.67 liters (1.32 vs. 2.00, 95%CI ; p=0.001) and by 0.55 liters (1.27 vs. 1.46, 95%CI ; p=0.002); respectively. Improvement in FEV1 levels was more prominent amongst patients with serum-IgE levels >600 kIU L–1. This change in FEV1 levels in 3 groups, was significantly different, as determined by one-way ANOVA (F (2,23)=7.959, p=0.002).

## Conclusion

In these patients add-on therapy with omalizumab improved lung function, quality of life and other clinical measures. In this small sub group analysis, the improvement was more marked in patients with a higher IgE level at baseline.

